# How fast is fisheries-induced evolution? Quantitative analysis of modelling and empirical studies

**DOI:** 10.1111/eva.12044

**Published:** 2013-01-24

**Authors:** Asta Audzijonyte, Anna Kuparinen, Elizabeth A Fulton

**Affiliations:** 1 Marine and Atmospheric Research, CSIROHobart, TAS, Australia; 2 Department of Environmental Sciences, University of HelsinkiHelsinki, Finland; 3 Department of Biosciences, Ecological Genetics Research Unit, University of HelsinkiHelsinki, Finland

**Keywords:** fisheries, mixed-model analyses, rate of evolution, rate of phenotypic change

## Abstract

A number of theoretical models, experimental studies and time-series studies of wild fish have explored the presence and magnitude of fisheries-induced evolution (FIE). While most studies agree that FIE is likely to be happening in many fished stocks, there are disagreements about its rates and implications for stock viability. To address these disagreements in a quantitative manner, we conducted a meta-analysis of FIE rates reported in theoretical and empirical studies. We discovered that rates of phenotypic change observed in wild fish are about four times higher than the evolutionary rates reported in modelling studies, but correlation between the rate of change and instantaneous fishing mortality (*F*) was very similar in the two types of studies. Mixed-model analyses showed that in the modelling studies traits associated with reproductive investment and growth evolved slower than rates related to maturation. In empirical observations age-at-maturation was changing faster than other life-history traits. We also found that, despite different assumption and modelling approaches, rates of evolution for a given *F* value reported in 10 of 13 modelling studies were not significantly different.

## Introduction

Contemporary evolution has recently gained a lot of attention among evolutionary and conservation biologists, with particular focus on human-induced phenotypic change in natural populations (Hendry et al. 2011; Palkovacs et al. 2012). Harvesting is one of the causes for such change and a likely driver of human induced evolution, as it typically targets individuals selectively in respect of some desired traits (Allendorf et al. 2008; Fenberg and Roy 2008; Darimont et al. 2009).

In fisheries, harvesting can greatly increase natural mortality and targets large, fastest growing and bold individuals (Heino and Godø 2002; Law 2007; Kuparinen et al. 2009). Life-history theory and experimental studies predict that such selective mortality will lead to slower growth, higher reproductive investment and maturation at younger ages (Stearns 1992; Law 2007; Walsh and Reznick 2008). Indeed, some of the earliest suggestions that fisheries-induced evolution (FIE) might be taking place came from observations of changing maturation schedules in heavily exploited North Atlantic species, such as cod and plaice (Law and Grey 1989; Rijnsdorp 1993; Olsen et al. 2004). In the past decade a number of experiments, theoretical modelling studies and empirical observations of the wild fish stocks have explored potential life-history consequences of size-selective harvesting (e.g. Conover and Munch 2002; Dunlop et al. 2009a; Sharpe and Hendry 2009). Overall, most studies largely agree in their qualitative predictions that size-selective harvesting will lead to an evolutionary response towards smaller body size and/or younger maturation age. Yet, there remain controversies as to whether the FIE rates are fast enough to represent a significant management concern (Jørgensen et al. 2007 and replies; Kuparinen and Merilä 2007; Andersen and Brander 2009).

Very fast rates of phenotypic change have been reported in some wild fish stocks and experiments. For example, the decline of age-specific maturation length for North Atlantic cod was about 10 cm in 7 years, representing about 3% change per year (Olsen et al. 2004). The decrease in weight-at-age obtained in experiments with Atlantic silversides was as high as ca. 40% in four generations (Conover and Munch 2002), whilst Jørgensen et al. (2007) reported harvest-induced evolutionary changes in wild fish stocks to be in the range of 20–30% over 13–125 years and called for an evolutionary impact assessment to be a standard practice in fisheries management. These views have been confronted by the ‘general evolutionary impact assessment’ presented by Andersen and Brander (2009), suggesting that FIE is slow and within the range of 0.1–0.6% per year; dealing with FIE is therefore less urgent than reducing direct declines in population sizes caused by overfishing.

A consistent comparison of rates of phenotypic change predicted by modelling studies and those detected in natural populations would be useful for an objective discussion about the management implications of FIE. In a recent summary of life-history changes in commercially exploited fish stocks, Sharpe and Hendry (2009) showed that most stocks exhibited some phenotypic trend consistent with the expected FIE direction and that the rate of this change was positively correlated with the fishing intensity. Andersen and Brander (2009) reported a 5–10 fold difference between the evolutionary rates estimated in their model and rates of phenotypic change observed in experiments and natural fish populations. Devine et al. (2012) compared rates of contemporary evolution in probabilistic maturation reaction norm midpoints of 26 exploited fish stocks. Most of their rates were in the range of 3–30 kilodarwins, depending on the fishing intensity; these rates correspond to 0.3–3.1% change per year. However, to our knowledge, there has been no consistent quantitative comparison and meta-analysis of different study types addressing FIE. In this review we investigate rates of FIE reported in empirical and modelling studies and ask three following questions: i) How fast are the rates of evolutionary and phenotypic change reported in theoretical modelling studies and empirical analyses of wild fish stocks, do they differ significantly between these two study types? ii) Do the rates of FIE differ among life-history traits? iii) Do different modelling studies predict significantly different rates of FIE?

## Methods

### Data collection

We screened literature for studies that reported the magnitude of evolutionary or phenotypic change in life history traits of fished stocks (real or modelled) occurring over a certain period of time. This excluded studies that: i) modelled final equilibrium conditions only and did not provide information about the rate of change (e.g. Baskett et al. 2005; Gårdmark and Dieckmann 2006; Arlinghaus et al. 2009; Vainikka and Hyvärinen 2012); ii) did not report the absolute value of phenotypic change of the trait studied or only reported changes in variance or residuals (e.g. Edeline et al. 2007, 2009; empirical part of the Hilborn and Minte-Vera 2008 study); iii) studies on phenotypic change in behavioural traits (e.g. Thériault et al. 2008), as a small number of such studies does not allow quantitative comparison (see Table S1 for included and excluded studies).

In all, this review included 14 modelling studies encompassing both analytical and stochastic individual-based eco-genetic models reporting 75 evolutionary rates, one review of empirical studies (Sharpe and Hendry 2009) and 14 original empirical studies not included in the Sharpe and Hendry (2009) review, reporting in total 147 rates of phenotypic change; in addition, two empirical studies where eight selection differentials were calculated from catch data were also included. Two modelling and one empirical study reported five data points of zero rates (Hilborn and Minte-Vera 2008; Wang and Höök 2009; and Devine and Heino 2011, see Table S1). Because our analyses required log-transformation of rates to achieve normality (see below), zero rates had to be excluded. In this way our meta-analyses compares rates of phenotypic change assuming that change does occur; cases where no change has been found cannot be included in the same statistical framework. This, and the fact that cases where no phenotypic change has been found are less likely to be reported in the literature, introduces a positive bias and should be taken into account when considering our findings (see Discussion). We also assessed 21 experimentally obtained evolutionary rates from Atlantic silverside experiments (Table S1), but did not include them in the meta-analyses because they either assessed evolutionary recovery with no size-selective harvesting or imposed much higher levels of fishing pressure than the empirical and modelling studies. Experimental rates are only used for comparative purposes. From the studies included in the meta-analyses, we recorded the magnitude of phenotypic or evolutionary change that took place in a given life-history trait, the period of time that was analysed, and the instantaneous fishing mortality *F* applied.

For the purpose of this study, we standardized the magnitudes of phenotypic or evolutionary change into percentage-of-change per year. Formal comparison of evolutionary rates should ideally use haldanes as a measurement unit. Yet, to calculate haldanes one needs trait variances and generation times, and these are currently available for too few populations included in the meta-analyses. Moreover, recent comparison of contemporary evolution rates in probabilistic maturation reaction norm midpoints of exploited fish stocks calculated in haldanes (scaled by generation) or darwins (scaled by year) revealed high correlation in the two estimates (Devine et al. 2012), suggesting that for the stocks in question scaling by generation or by year leads to similar conclusions. The simple statistics of percentage-of-change per year is also preferable due to its clarity for nonevolutionary biologists and direct applicability in ecological and fisheries management contexts. The direction of phenotypic change differed for different life-history traits, e.g. maturation age decreased with fishing pressure whereas reproductive capacity increased. Here, only the absolute value of the rate was used, as we investigated the magnitude of the effect of fishing on the rate of phenotypic change. The list of studies, together with the data and assumptions made to standardize the results are given in the Table S1.

Life-history traits reported in the studies reviewed here were grouped into the following five categories: size at maturity (SZM), age at maturity (AGM), midpoint of the probabilistic maturation reaction norm (PMN), growth traits (GRO) and reproductive investment (REP) (Table S1). In some studies, the magnitude of fishing was only reported approximately (high or low); for these studies an arbitrary fishing level was assumed (*F* = 0.2 per year for low, 0.7 for high, 0.8–1.0 for very high and exceeding 1.0, see Table S1 for further details). We repeated all analyses without such studies to assess the robustness of the results to the arbitrary assumed fishing level. Two studies reported only selection differentials (*S*) but not the rate of phenotypic change. For these studies an evolutionary response (*R*) was calculated using the breeders equation (*R = S·h*^2^), assuming heritability at *h*^2^ = 0.3 (e.g. Mousseau and Roff 1987). Clearly such calculations are not accurate, as the breeder's equation does not fully apply for overlapping generations, the estimated *S* may not integrate all the selection up to reproduction, and the exact value of heritability is debatable. As in the case of uncertain fishing rates, analyses were repeated with the latter studies excluded (incidentally, studies reporting selection differentials only also did not have information on accurate fishing rates). Another problem for quantitative comparison among different studies is the variability of assumptions made in different models. To account for possible associations among the rates observed within the same modelling study and for the uneven number of observations within different studies, we treated the study ID as a random effect in the analyses. Finally, it must be emphasized that phenotypic change in the wild, as reported in empirical studies, can be caused by a range of factors other than evolution and therefore cannot be strictly compared with evolutionary rates reported in modelling or experimental studies. We nevertheless conduct such a comparison to explore the overall trends in the rate of phenotypic changes, as well as to quantify how much they differ from rates projected by the modelling studies. Fishing rate *F* is reported as rate per year.

### Analysis

To first illustrate the overall patterns in the rate of evolutionary or phenotypic change, we plotted the rate of change against fishing pressure (*F*) for the two study types (modelling and empirical) and fitted simple least-square fit regression lines to show the correlations. We then continued by analysing the data using linear mixed-effect models. In these models the study ID was treated as a random effect whereas fishing pressure (*F*), trait group (*TRAIT*) and study setup (*SS*; empirical or modelling) were treated as fixed effects (*F* as continuous, and *TRAIT* and *SS* as factors). The response variable, i.e. the rate of phenotypic change (*R*), was log-transformed for the sake of normality and homogeneity of residuals. To allow for the log-transformation of the response, we needed to exclude four data points from two modelling studies in which the reported *R* was zero (Hilborn and Minte-Vera 2008 and Wang and Höök 2009 studies, see Table S1).

Firstly, to assess the overall rates of phenotypic change, their dependence on *F*, and differences between empirical and modelling studies we fitted a model (Model 1)


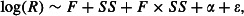
(1)

where *α* is the random effect of the study ID, *ε* is the normally distributed error term and × indicates two-way interaction between *F* and *SS*. The random effect *α* is used to account for unbalanced numbers of observations of different traits in empirical and modelling studies and allow for variation in *R* around a common intercept associated with different traits.

To investigate whether the rates of phenotypic change differ among different life-history traits, we fitted the following model to data from the empirical and modelling studies separately (Models 2 and 3):


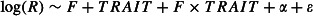
(2)

Both models were fitted by maximum-likelihood method and reduced stepwise using likelihood-ratio comparisons with *χ*^2^ statistics as suggested by Crawley (2007). All the analyses were first performed with the full data set and then excluding the observations, where *R* was estimated based on *S* and where *F* was only approximately known.

To investigate whether different modelling studies reported significantly different evolutionary rates we grouped modelling studies into eight groups or study types. The grouping was done based on the similarity of assumption used in the models: (1) eco-genetic models of Dunlop et al. (2007, 2009a,b), Enberg et al. (2009) and Okamoto et al. (2009), (2) eco-genetic model of Wang and Höök (2009), (3) models of Andersen et al. (2007) and Andersen and Brander (2009), (4) individual-based model of Brown et al. (2008), and four remaining models where (5) is for de Roos et al. (2006), (6) for Hard et al. (2009), (7) for Eldridge et al. (2010) and (8) for Kuparinen and Hutchings (2012). For this data set we fitted a linear model (Model 4):


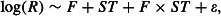
(3)

where *ST* is the study type. For this model we do not treat individual studies as a random effect, because most study types included only one study. All the analyses were performed using R 2.12.1 (R Development Core Team 2010) and residuals were checked for normality and homogeneity.

## Results

### Rates of fisheries-induced phenotypic change

**Figure 1:**
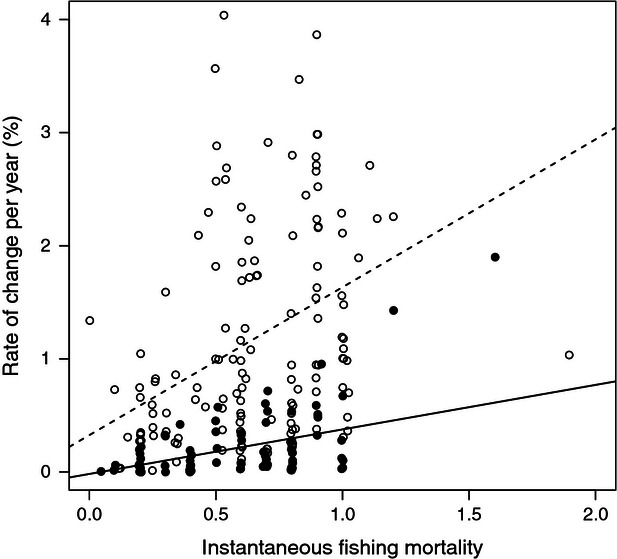
Rates of phenotypic change reported in modelling (black) and empirical (white) studies. For clarity data points are slightly jittered on the *x*-axis. Least-square fit regression lines were fitted to modelling (solid line) and empirical (dashed line) studies separately. Note that regression lines in the figure are fitted to raw rate values and therefore differ from those in the mixed-model analyses (fitted to log-transformed rates).

There was considerable variation in the rates of evolutionary change reported in modelling studies; for example, for *F* values ranging from 0.5 to 1.0 per year, rates of evolution varied from 0.02% to 0.93% per year, with the mean of 0.25% per year (Fig. 1). Similarly, for the same magnitude of fishing pressures, rates of phenotypic change reported in empirical studies ranged from 0.11% to 4.04% per year, with the mean value of 1.10% per year. Evolutionary rates reported in experiments, where fishing pressure was extremely high (*F* = 2.3 corresponding to 90% of individuals taken out every generation) ranged from 1.15% to 17.38% per year. One data point reporting rate of 6.94% per year at *F* = 2.3 fishing value from the modelling study of Brown et al. (2008) was obtained by modelling the experimental setup of Conover and Munch (2002). This data point fitted well into the range of evolutionary rates observed in experiments, but was a clear outlier in rates and *F* values among the modelling and empirical studies and had disproportional influence on the regression in the mixed-model analyses; this data point was therefore removed from further analyses of empirical and modelling data.

In the first mixed-model analysis (Model 1 in Table 1) we explored the effects of *F* on the study setup, SS: empirical or modelling (Formula 1). Overall, the log of phenotypic change, log(*R*), was positively and significantly correlated with the fishing pressure, yet the interaction between *F* and SS was not significant and could be removed (*χ*^2^ = 1.27, df = 1, *P* = 0.26). This suggested that the effect of *F* on the rate of phenotypic change, i.e. the slope, did not significantly differ between modelling and empirical studies. However, the intercepts differed between empirical and modelling studies, which means that, for a given value of *F*, the magnitude of change was larger in empirical than in modelling studies (the coefficient for modelling studies was = −1.44) (Table 1). Exclusion of data points with uncertain *S* and *F* values (*n* = 29) gave very similar results in terms of the significant covariates and their coefficients (Intercept = −1.37, *F* = 1.81, *SS* = −1.68).

**Table 1:** Effects of significant covariates on the log-transformed rate of phenotypic change, as estimated through fits of linear mixed-effect (Models 1–3) or general linear (Model 4) models

Model term value	Coefficient (SE)	*χ*^2^ or F*	*P*-value
Model 1: effects of *F* and study setup in modelling and empirical studies combined (*n* = 216)			
Intercept (EMP†)	−1.59 (0.28)		
*F* (EMP)	1.79 (0.245)	49.56 (df = 1)	<0.001
*SS*: MOD†	−1.44 (0.33)	14.06 (df = 1)	<0.001
Model 2: effects of *F* and trait in modelling studies only (*n* = 70)			
Intercept (GRO + REP‡)	−4.10 (0.39)		
*F* (GRO + REP)	2.32 (0.38)	34.45 (df = 1)	<0.001
*TRAIT* levels: (PMN + SZM + AGM‡)	1.30 (0.29)	17.88 (df = 1)	<0.001
Model 3: effects of *F* and trait in empirical studies only (*n* = 146)			
Intercept (AGM)	−0.40 (0.39)		
*F* (AGM)	0.65 (0.44)		
*TRAIT* levels: (PMN + SZM + GRO)	−1.41 (0.37)		
*TRAIT* levels: REP	−4.99 (1.72)	14.01 (df = 2)	<0.001§
*F* × (PMN + SZM + GRO)	1.54 (0.54)	9.67 (df = 2)	0.008¶
*F* × REP	4.34 (2.16)		
Model 4: effects of study type in modelling studies (*n* = 70)			
Intercept (Models-I**)	−2.98 (0.26)		
*F*	2.42 (0.38)	40.27 (df = 1)	<0.001
*ST* levels: Models-II**	−1.73 (0.23)	55.54 (df = 1)	<0.001

*F*, instantaneous fishing mortality; *SS*, study setup; *TRAIT*, phenotypic trait group; *ST*, modelling study type (see Methods).

* *χ*^2^ statistics of likelihood ratio test used to compare linear mixed-effect models and *F* statistics used for the general linear models.

† Study setup: empirical (EMP) and modelling (MOD).

‡ Traits: probabilistic maturation reaction norm traits (PMN), size at maturity (SZM), age at maturity. (AGM), growth traits (GRO), reproductive investment (REP).

§ values for joining REP with PMN + SZM + GRO.

¶ values for removing both *F* × (PMN + SZM + GRO) and *F* × REP interactions at once.

** Two groups of modelling studies: (1) + (3) + (4) + (5) + (8) (Models-I) and (2) + (6) + (7) (Models-II) (see Methods for the list of models and references).

### Comparing rates of phenotypic change in different life-history traits

**Figure 2:**
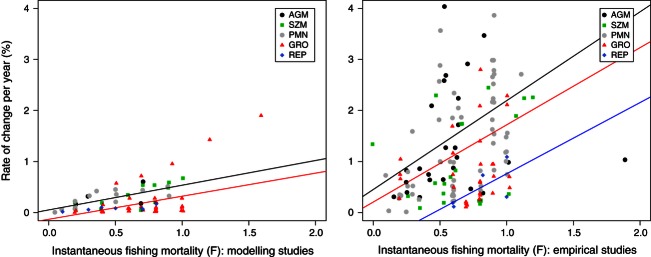
Rates of phenotypic change for five trait types as reported in empirical and modelling studies. Trait types: age at maturity (AGM), size at maturity (SZM), midpoint of the probabilistic maturation reaction norm (PMN), growth traits (GRO) and reproductive investment traits (REP). Least-square fit regression lines were fitted to the groups of traits that differed significantly in the linear model analyses (Table 1); for the regression fit one outlier data point *F* > 1.5 was removed from each of modelling and empirical data sets. Note that regression lines in the figure are fitted to raw rate values and therefore differ from those in the mixed-model analyses (fitted to log-transformed rates).

In the mixed-model analyses of the modelling studies (Model 2, Formula 2), the interaction between *F* and trait could be removed (*χ*^2^ = 0.35, df = 4, *P* = 0.97), suggesting that responses to increasing *F* values did not differ significantly among traits. In the stepwise model reduction, the five trait groups – reproductive investment (REP), growth (GRO), age at maturation (AGM), size at maturation (SZM) and values of probabilistic maturation reaction norms (PMN) could be combined into two trait groups REP + GRO and PMN + SZM + AGM (model reduction steps: GRO + REP: *χ*^2^ = 0.02, df = 1, *P* = 0.90; PMN + AGM: *χ*^2^ = 0.17, df = 1, *P* = 0.68; PMN + SZM + AGM, *χ*^2^ = 1.86, df = 1, *P* = 0.17). As in the previous analyses, fishing intensity significantly increased the rate of phenotypic change, with PMN + AGM + SZM traits evolving faster than GRO + REP traits for a given value of *F* (Table 1, Fig. 2).

In contrast to the modelling studies, the mixed-model analysis of empirical studies (Model 3, Formula 2) showed that the interaction between *F* and *TRAIT* could not be removed (*χ*^2^ = 12.22, df = 4, *P* = 0.02). In the stepwise model reduction, GRO and SMZ could be joined into one factor level (*χ*^2^ = 0.40, df = 2, *P* = 0.82), and the combined GRO + SZM trait could be joined with PMN (*χ*^2^ = 3.81, df = 2, *P* = 0.15). After these trait levels were combined, the *F* × *TRAIT* interaction still could not be removed (Table 1). In the final model the intercept was lowest for REP and highest for AGM and the rate of change increased significantly with increasing *F*, but the magnitude of increase varied among traits. The slope (i.e. the rate of increase in the rate with increasing *F* values) was lowest for AGM (0.65) and highest for REP (0.65 + 4.34) (Table 1, Fig. 2).

The analyses of the empirical studies were repeated after excluding the data points with unknown *F* and *R* (*n* = 29). The mixed-model analyses results of this reduced data set were similar in that GRO, PMN could be combined with SZM (*χ*^2^ = 0.72, df = 1, *P* = 0.70 and *χ*^2^ = 2.85, df = 1, *P* = 0.24), but neither AGM nor REP could be combined with GRO + SZM + PMN group (*P* = 0.001 for both cases). The coefficients in the final model were similar to that with all data points included (Intercept = −0.15, *F* = 0.64, *TRAIT* level GRO + SZM + PMN = −1.47, *TRAIT* level REP = −4.98, interaction *F* × GRO + SZM + PMN = 1.69 and interaction *F* × REP = 4.31).

### Comparing rates of phenotypic change in different modelling studies

**Figure 3:**
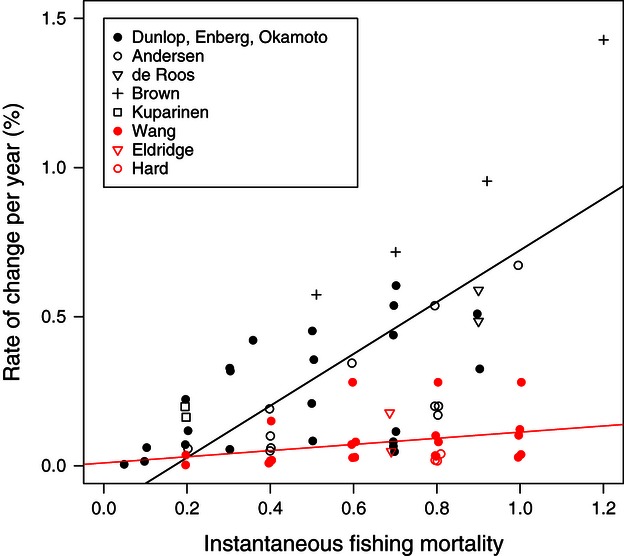
Rates of evolutionary change reported in different modelling studies. Two least-square fit regression lines were fitted to the two groups of models that differed significantly in the linear model analysis (Table 1). The eight model groups are presented in the legend by the name of the first author (see Methods for full references).

In the model that compared rates between different modelling studies (Model 4, Formula 3), the interaction between *F* and the study type was not significant (*F* = 0.50, df = 3, *P* = 0.69) and could be removed. Rates of phenotypic change in eco-genetic models of Dunlop et al. (2007, 2009a,b), Enberg et al. (2009) and Okamoto et al. (2009) (type 1) did not differ from those in the models of Andersen et al. (2007) and Andersen and Brander (2009) (type 3) (*F* = 0.19, df = 1, *P* = 0.67). They also did not differ from rates in the model of Brown et al. (2008) (type 4) (*F* = 3.09, df = 1, *P* = 0.08), from rates in de Roos et al. (2006) study (type 5) (*F* = 0.07, df = 1, *P* = 0.79) and from rates in Kuparinen and Hutchings (2012) (type 8) (*F* = 1.74, df = 1, *P* = 0.19). These five model types could be joined into one larger category, further called Models-I. Likewise, rates in the Wang and Höök (2009) eco-genetic model (type 2) did not significantly differ from those in the Eldridge et al. (2010) study (type 7) (*F* = 0.76, df = 1, *P* = 0.39), and from those in the study of Hard et al. (2009) (type 6) (*F* = 3.96, df = 1, *P* = 0.05) so the three study types were combined into one category, called Models-II. The final model showed that for all models *F* had a significant and positive effect on the rates of evolutionary change, but for a given value of *F* evolutionary rates in Models-II were slower than in Models-I (Table 1, Fig. 3). The Fig. 3 shows two regression lines fitted for the two corresponding categories using the least-square fit, where coefficient of determination values were *R*^2^ = 0.68 (df = 41, *P* < 0.001) for Models-I and *R*^2^ = 0.47 (df = 25, *P* = 0.012) for Models-II.

## Discussion

Our quantitative comparison of phenotypic change rates reported in empirical studies of wild fish stocks and in theoretical models gave four main findings. First, the rates in empirical studies are roughly four times faster than those in theoretical models. Second, despite this difference in rates, the correlation between the fishing intensity and the rate of change is very similar in the two groups of studies, suggesting that fishing is responsible for some of the phenotypic change observed in wild stocks. Third, life-history traits related to maturation, and especially age-at-maturation, appear to change faster than traits related to growth and reproductive investment. Fourth, despite different assumptions and modelling approaches, ten of thirteen modelling studies included in our meta-analysis, yielded similar rates of FIE.

### Empirical phenotypic rates are about four times faster than modelled evolutionary rates

Fast rates of contemporary phenotypic change are seen in many environments and often seem to be caused by human influence such as harvesting, habitat disturbance or introduction of new species (Hendry et al. 2008, 2011; Darimont et al. 2009). Yet, it remains unclear how much of the change observed in wild population is caused by evolution. Hendry et al. (2008) found that rates of phenotypic change in human-affected environments were 1.7 times faster than those in natural environments, but most of this change was considered to be caused by phenotypic plasticity. Despite advances in evolutionary biology it remains notoriously difficult to invoke evolution as an explanation for phenotypic changes in wild populations (Merilä et al. 2001) and wild fish are no exception (Swain and Foote 1999). Statistical approaches, such as probabilistic maturation reaction norms (PMRN) (reviewed in Heino and Dieckmann 2008), and mixed-effect or general linear models (Edeline et al. 2007, 2009; Swain et al. 2007) have been used to disentangle impacts of environmental change from potential evolutionary responses. However, even these approaches may miss some potential factors such as changing temperature, food or habitat or maternal environmental effects that could explain observed phenotypic changes without having to invoke evolution (Morita et al. 2005; Kuparinen et al. 2011; Uusi-Heikkilä et al. 2011; Salinas and Munch 2012). Strictly speaking, unequivocal evidence for adaptive evolution can only be obtained by demonstrating that the suspected selective factor is causing genetic changes in the population (shown through direct DNA analyses or common-garden experiments) (Kuparinen and Merilä 2007; Hansen et al. 2012). However, to date very few studies have managed to do so. One of the best examples is the study of Atlantic cod by Jakobsdóttir et al. (2011), demonstrating no temporal change in gene frequencies of neutral markers but a change in a gene under selection. The selected gene (*Pantophysin*) was correlated/responsible for differences in size-at-age and schooling behaviour of cod, and alleles responsible for more offshore schooling started disappearing after the introduction of offshore fisheries. Yet, even in this case there was no direct evidence that fishing preferentially selects the disappearing genotypes and alternative explanations for the change in gene frequencies could not be completely ruled out.

In our meta-analysis we find that the empirically detected phenotypic rates are about four times faster than the theoretically predicted evolutionary rates, but that the overall correlation between fishing intensity and the rate of change is very similar in the two sets of studies. This adds weight to the hypothesis that evolutionary changes are responsible for a part of the phenotypic change observed in the wild and that fishing is the primary driver of such changes. However, the difference in the rates of change observed between theoretical and empirical studies suggests that either a large proportion of the phenotypic change observed empirically is caused by plasticity (e.g. Hendry et al. 2008) or that modelling studies consistently underestimate the strength of fisheries-induced selection. In wild, numerous environmental variables can modify life-histories in ways that are not accounted for in the modelling studies. For example, increasing temperature can advance maturation, further strengthening the fishing-induced evolutionary changes. Environmental maternal effects can plastically reduce growth and adult size of juveniles – if the spawning stock consists of young, small fish that managed to escape fishing, their offspring is likely to be smaller and have slower juvenile growth (Salinas and Munch 2012; this is regardless of the final fitness of the offspring, see Marshall et al. 2010). However, it is also possible that fisheries in wild stocks are imposing considerably stronger selection than what is assumed in models, by selecting against behavioural traits. Biro and Post (2008) showed fast depletion of bold behaviour genotypes in experimentally designed gillnet fishery. The change in *Pantophysin* gene allele frequencies observed in Atlantic cod was also correlated to behaviour, such as feeding and schooling depth (Jakobsdóttir et al. 2011). In fact, the gene was correlated to larger size-at-age, which could also be achieved through more aggressive behaviour. More experimental and modelling study is needed to explore impacts of FIE on fish behaviour. Notably in the eco-genetic model of Dunlop et al. (2007) introduction of behavioural traits such as parental care slowed down rather than increased modelled evolutionary rates.

In summary, while theoretical models cannot incorporate a multitude of factors that might affect phenotypic rates in wild stocks, it is nonetheless encouraging that different modelling approaches broadly converge in their predictions about rates of fisheries-induced evolution. In most modelling studies rates are similar and on average at 0.2% and 0.4% of evolutionary change per year for *F* = 0.4 and *F* = 0.6 respectively. This translates to 10% and 18% of evolutionary change in 50 years.

### Study period as a possible factor explaining difference in rates of phenotypic change

One factor that could partly explain the difference in phenotypic rates between study types and traits is the length of the study period. If a population has reasonable amount of genetic variance in a life-history trait, its initial response to selection will be fast. As genetic variation gets depleted by selection evolutionary response will slow down. As a result, evolutionary rates measured over short time scales are usually faster than if measured over long-time periods. In our meta-analyses most experimental rates are measured over a period of few generations, empirical data are typically collected over less than 50 years, whereas modelling studies mostly model evolution over 100 years or more (See Fig. S1). The length of the study period is therefore likely to be one of the reasons why experimental rates are much faster than empirical rates (see below). The same could apply to the comparison of empirical and modelling studies, although modelling studies typically assumed constant values of genetic variance and therefore modelled evolutionary rates were rather constant over time. We could not address the importance of the study period statistically because the study lengths were too different for modelling and empirical studies (Fig. S1). Only 20 data points from five studies were obtained by modelling time periods shorter than 100 years and they reported both some of the fastest and slowest evolutionary rates (Table S1). We suggest that future modelling attempts could be designed to explore the importance of the study period on the evolutionary rates, or at least conduct simulations over the time scales comparable with those in empirical studies.

### Why are the experimental evolutionary rates so fast?

Some of the fastest rates of harvest-induced evolution have been described from common-garden experiments with Atlantic silversides (Conover and Munch 2002; Walsh et al. 2006). Egg volume, size-at-hatch and length-at-age in experiments harvesting large specimens decreased by 0.9–2.6% per year, whereas growth efficiency and weight-at-age of 190 days decreased by staggering six and 17% per year respectively. For comparison, fastest empirical rates included in our study were within the range of 3.5–4.0% per year and applied to the maturation ages of Atlantic cod. Fastest evolutionary rates in modelling studies were 1.4–1.9% and applied to very high fishing intensities (*F* = 1.2–1.6) modelled in Brown et al. (2008). There are at least four reasons that might explain why experimental rates are so high and very different from findings in empirical and modelling studies. First, generation times of animals used in experimental studies are several-fold shorter than that for most wild stocks. As we report phenotypic change by year rather than per generation, animals with shorter generation times will have faster rates. Second, experiments are conducted on time scales of several generations. Large amounts of genetic variance available in the initial wild population leads to fast evolutionary response, introducing the time dependency of evolutionary rates, discussed above. Third and possibly the main reason is that experiments used extreme harvest conditions, with e.g. 90% of largest individuals taken out with ‘knife-edge’ precision at every generation in Conover and Munch (2002) study. Such harvesting regime is unrealistic for real fisheries (Brown et al. 2008; Hilborn and Minte-Vera 2008; Andersen and Brander 2009). Brown et al. (2008) compared selection applied in Conover and Munch (2002) experiments to that in realistic fisheries and showed that evolutionary rates in wild stocks are expected to be 2.5–5 times slower than in experiments. Likewise, Hilborn and Minte-Vera (2008) demonstrated that the size selectivity in experiments is unrealistically strong compared with the real fisheries of Atlantic cod (Fig. 1 in Hilborn and Minte-Vera 2008). In fact, they found no evolutionary response in growth rate when modelling fisheries of cod under realistic harvest regimes (but see below on the evolution of growth). The fourth possible reason for fast phenotypic rates in experiments is that common-garden experiments confound evolutionary and maternal effects (Swain and Foote 1999). As only the smallest females get a chance to reproduce, they will spawn small eggs which, when raised in common garden experiment conditions will grow into small fish (Marshall et al. 2010; Salinas and Munch 2012). Such maternal effects could also explain the rapid experimentally obtained recovery of length-at-age (ca. 1.5% per year; Conover et al. 2009) once fishing was ceased. Yet, it should be noted that experiments with guppies introduced to predator-free environments gave somewhat similar rates of phenotypic recovery (0.5–2.7% per year; Reznick et al. 1997) (Table S1).

### Traits related to maturation might be evolving faster than traits affecting growth

Our meta-analysis suggests that rates of phenotypic change differed among life-history traits (Fig. 2). In modelling studies, traits related to maturation (PMN, SZM and AGM) changed faster than growth and reproductive traits, whereas in empirical studies age-at-maturation (AGM) changed faster than the other traits. Thus, both types of studies found growth and reproductive investment to be among the slowest evolving traits. The four data points from modelling studies that found zero evolutionary rate and were excluded from the formal mixed-model analyses were all related to growth rate (Table 1). A notable exception is the empirical study of Nusslé et al. (2011) study reporting 2.1–2.8% chance in logarithmic growth rates of *Coregonus* fish is lakes (Table S1 and fastest rates of GRO in Fig. 2). In fact, Hilborn and Minte-Vera (2008) did not find any support for reduced growth rates in their meta-analysis of 73 commercially fished stocks.

On the one hand it may seem that growth rate indeed evolves very slowly, either due to lower heritabilities or weaker fisheries imposed selection. Natural selection on growth or size in early life stages might be strong enough to counter the evolutionary pressure imposed by fisheries (Edeline et al. 2007, 2009; Perez and Munch 2010). Also any evolutionary decrease in growth rates might be hidden by the plastic increase due to reduced population density and competition for food. However, several cautionary notes have to be made. Firstly, in modelling studies growth (GRO) was typically studied on longer time scales than other traits, which could possibly introduce the time dependency of evolutionary rate discussed above (Fig. S1). Study periods for different traits were more evenly distributed for empirical studies (Fig. S1) and we conduced additional mixed-model analyses to assess the effect of study period on the rate of change. We found that the study period was not a significant explanatory factor of phenotypic rate (*P* = 0.10), but this could also be due to the lack of statistical power. Notably, study periods of reproductive investment (REP) were more similar to that of other traits in both modelling and empirical studies (Fig. S1), suggesting that length of the study period is not likely to be a main reason for the difference in rates. Secondly, many empirical studies have investigated potential FIE in maturation schedules and probabilistic reaction norms (Dieckmann and Heino 2007), whereas changes in growth are typically assumed to be plastic and empirical studies looking at FIE in growth remain rare. A recent review by Enberg et al. (2012) has highlighted the difficulties in disentangling the effects of fisheries on growth, pointing out that even the expected direction of fisheries-induced growth rate evolution, i.e. increase or decrease, is not immediately obvious, and can change depending on a range of factors. This could be one reason why the meta-analysis of Hilborn and Minte-Vera (2008) found no evidence for reduced growth rates when rates over a large range of fish stocks were pooled. In empirical studies, growth rate is typically inferred from size-at-age data and the latter is determined by the combination of growth rate, maturation age and reproductive investment, making inferences about growth rate itself difficult (Enberg et al. 2012).

### Concluding remarks

Contemporary evolution and human-induced trait changes are rapidly developing fields of wildlife conservation (Stockwell et al. 2003) and fisheries research (Sharpe and Hendry 2009). The present meta-analysis provides a synthesis about rates of phenotypic changes observed in fish in the wild and those predicted by theoretical models. Clearly such broad meta-analyses combines results from studies with different assumptions and suffers from positive literature bias (studies where no phenotypic change has been found are less likely to be reported) and positive statistical bias (five data points where no evolution has been found were excluded from the statistical analyses). Nevertheless, our findings provide some insights into which traits might be expected to change in harvested fish population and how fast. They also point to areas where more research is needed. First, future modelling studies should explore the time dependency of phenotypic rates and be conducted on timescales comparable to the observations from wild stocks. Second, growth rate and reproductive investment appears to be among the slowest evolving traits, but more data are needed to disentangle the confounding effects of growth, maturation and reproductive investments. Third, very little is known about the strength of fisheries-induced selection on behavioural traits that can affect life-history evolution. We hope the results of this study will contribute to and stimulate further discussion and investigation into the rates and ecological importance of contemporary evolution.

## Data archiving statement

Data used in these analyses are provided in the Electronic Supplement Table S1.

## Supporting Information

**Figure S1.** Boxplots of the study durations for different study types and traits

**Table S1.** Data used in the analyses
