# Supplementary figures and images for: How fast is fisheries-induced evolution? Quantitative analysis of modelling and empirical studies
Source: Evol Appl. 2013 Jan 24;6(4):585–95. doi: 10.1111/eva.12044 (PMC3684740; doi:10.1111/eva.12044)

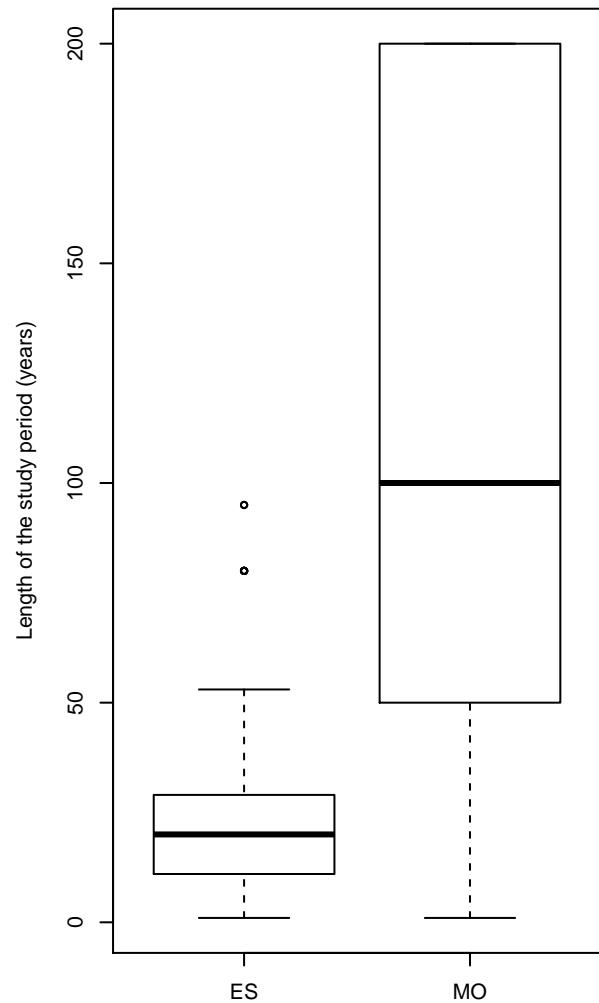

Empirical vs. modelling studies

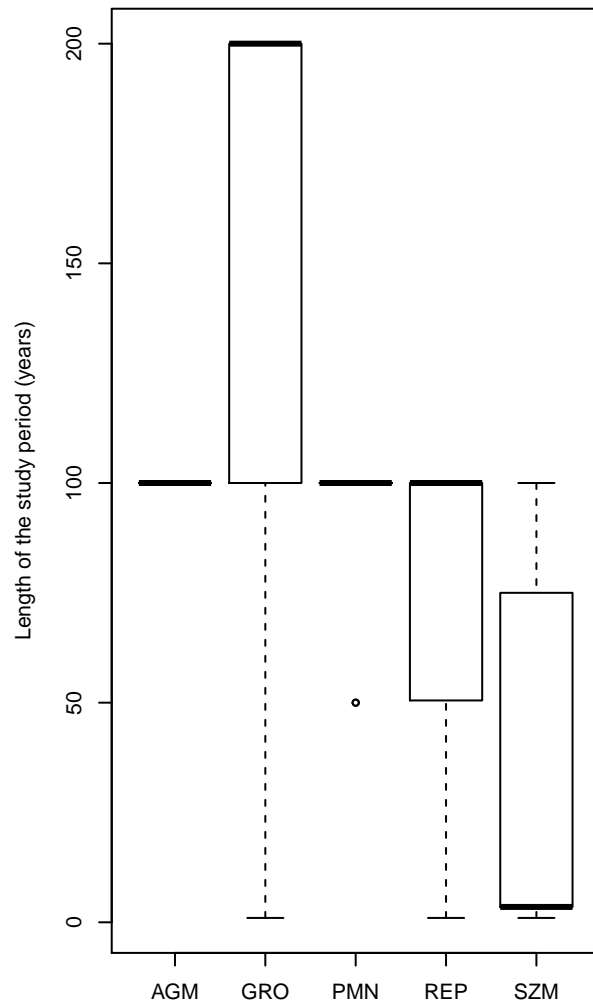

Traits: modelling studies

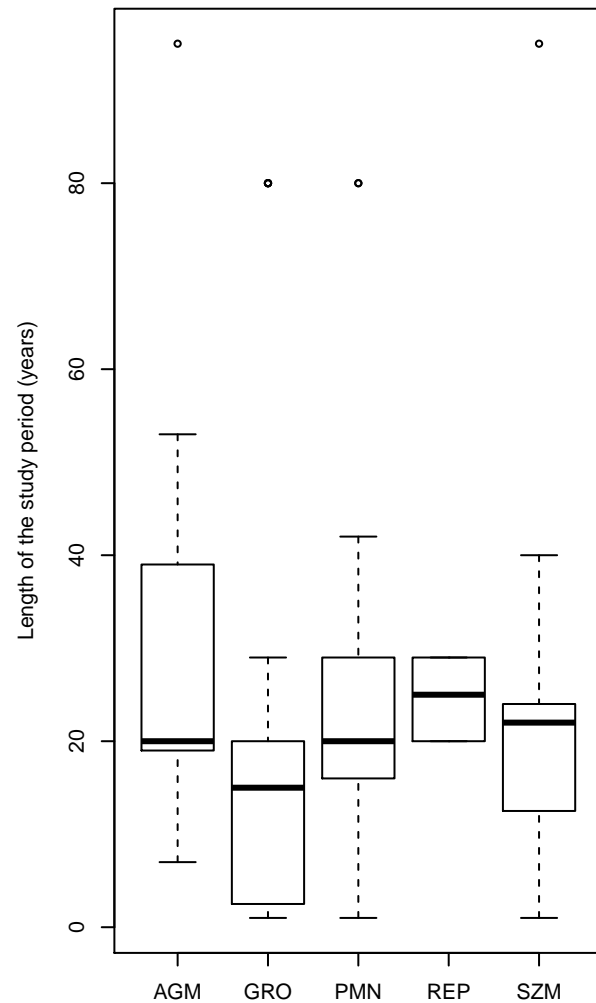

Traits: empirical studies

Supplement: Supplementary file 1 [file eva0006-0585-SD1.pdf]
